# Mogroside‐Rich Monk Fruit Extract Improves Glycemic Control Without Promoting Additional Weight Gain Compared With Sucralose and Sucrose in High‐Fat Diet‐Induced Obese Mice

**DOI:** 10.1002/fsn3.72121

**Published:** 2026-07-14

**Authors:** Jessika Woo Kar Man, Yu‐Cih Huang, Rong‐Hong Hsieh, Sassy Bhawamai, Yue‐Hwa Chen

**Affiliations:** ^1^ School of Nutrition and Health Sciences, College of Nutrition Taipei Medical University Taipei Taiwan; ^2^ CPF Food Research and Development Center Co., Ltd Phra Nakhon Si Ayutthaya Thailand; ^3^ School of Food Safety, College of Nutrition Taipei Medical University Taipei Taiwan

**Keywords:** glucose tolerance, mogroside, monk fruit extract, non‐nutritive sweeteners, obesity, sucralose

## Abstract

A high‐fat diet (HFD) with excessive sugar intake contributes to obesity and type 2 diabetes, leading to increased use of non‐nutritive sweeteners (NNS). However, evidence on their metabolic effects remains inconsistent, especially between natural and synthetic NNS. This study compared the effects of a natural NNS, monk fruit extract (MFE), with sucrose and synthetic sucralose, at equivalent sweetness on body weight and glucose homeostasis in HFD‐induced obese mice. Male C57BL/6 mice were fed an HFD for 8 weeks to induce obesity, then randomized to receive distilled water (control), sucrose (266 g/L), MFE (1.1 g/L; ~50% mogroside V), or sucralose (0.36 g/L) in drinking water for another 8 weeks. Body weight, food and water intake, and oral glucose tolerance tests (OGTT) were assessed. At sacrifice, serum biochemistry, organs, tissues, and duodenal expression of sweet taste receptors (T1R2/T1R3) and glucose transporters (SGLT‐1/GLUT2) were analyzed. The results showed that sucralose and sucrose further increased body weight gain, whereas MFE did not promote additional weight gain despite increased food intake. MFE significantly reduced fasting blood glucose, while sucralose impaired glucose tolerance, reflected by increased OGTT area under the curve. No significant differences in visceral adipose tissue weights or duodenal T1R2, T1R3, SGLT‐1, and GLUT2 expression were observed among groups. In conclusion, MFE, within ADI‐equivalent doses, improved fasting glycemia without promoting further weight gain compared with the HFD‐control, whereas sucralose exacerbated obesity‐related impaired glucose tolerance. These findings suggest that natural sweeteners such as MFE may represent a safer alternative to added sugars and synthetic NNS for weight management and glycemic control for obese subjects.

## Graphical abstract

This study investigated the effects of natural monk fruit extract (MFE), synthetic sucralose, and sucrose at equivalent sweetness levels within the acceptable daily intake (ADI) range on body weight, adiposity, and glucose homeostasis in HFD‐induced obese C57BL/6 mice. The results demonstrated that MFE, within ADI‐equivalent doses, improved fasting glycemia without promoting further weight gain compared with the HFD‐control, whereas sucralose exacerbated obesity‐related impaired glucose tolerance.

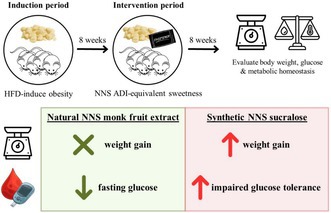

**Abbreviations**
ACE‐K: acesulfame potassium
ADI: acceptable daily intake
ALT: alanine aminotransferase
AST: aspartate aminotransferase
AUC: area under the curve
GLUT2: glucose transporter 2
HDL‐C: high‐density lipoprotein cholesterol
HFD: high‐fat diet
LDL‐C: low‐density lipoprotein cholesterol
MFE: monk fruit extract
NNS: non‐nutritive sweetener
OGTT: oral glucose tolerance test
SGLT‐1: sodium‐dependent glucose co‐transporter‐1
STR: sweet taste receptor
T1R2/T1R3: taste receptor subunits T1R2 and T1R3
TC: total cholesterol
TG: total triglycerides

## 1 Introduction

The consumption of high‐fat diets (HFDs) combined with excessive sugars, such as sucrose or corn syrup, has been strongly associated with obesity, type 2 diabetes, insulin resistance, and dyslipidemia (Unger and Orci 2001). To address these concerns, non‐nutritive sweeteners (NNS) are widely used as sugar substitutes to reduce caloric intake and support blood glucose control, particularly in individuals with obesity or diabetes. NNS can be broadly classified as synthetic (e.g., aspartame, acesulfame potassium [ACE‐K], sucralose) or natural (e.g., stevia, licorice root extract, monk fruit extract [MFE] (Trayhurn 2013)). However, safety concerns remain, particularly for synthetic NNS, as reflected by the recent designation of aspartame as “probably carcinogenic to humans” by the International Agency for Research on Cancer of the World Health Organization (WHO 2023, July 14).

MFE, extracted from *Siraitia grosvenorii* (Luo Han Guo), was approved by the USDA in 2020 as a natural sugar substitute and has gained considerable attention in the food industry due to it contains no calorie, favorable taste profile and lack of bitter aftertaste. It is now applied in a wide range of products, including beverages, juices, dairy foods, confectioneries, and dietary supplements. The sweetness of MFE is primarily derived from mogrosides, a class of terpene glycosides, with mogroside V being the most abundant. Mogroside V exhibits 150–250 times the sweetness intensity of sucrose, and commercial MFE powders typically contain 25%–50% of this compound (Muñoz‐Labrador et al. 2021). It has been indicated that ingested mogrosides are mainly degraded by digestive enzymes and gut microflora, and excreted as mogrol and its glucosides (Murata et al. 2010). Mogroside V can be also detected in blood, organs, and urine in rats fed with purified mogroside V for 3 days (Xu et al. 2015), indicating intact mogroside V can also be absorbed. Evidence from human and animal studies suggests potential metabolic benefits. In healthy adults, a single dose of MFE (0.63 g, containing 50% mogroside V) did not alter blood glucose or insulin levels compared to sucrose (65 g) (Tey et al. 2017). In contrast, an 8‐week supplementation with higher doses of mogroside V (100 mg/kg/day) reduced body weight and blood glucose levels in HFD‐induced obese mice (Li et al. 2020), while MFE‐sweetened yogurt (15~100 mg/kg/day) improved fasting blood glucose and HOMA‐IR in diabetic rats compared with sucrose‐sweetened yogurt after 6 weeks of consumption (Ban et al. 2020). Collectively, these findings suggest that MFE may contribute to regulating body weight and glucose homeostasis.

Blood glucose regulation is primarily governed by the insulin‐glucagon axis, in which insulin lowers postprandial glucose, while glucagon increases glucose levels during fasting. In addition, sweet taste receptors (STRs) have been implicated in glucose homeostasis. STRs are heterodimeric G protein‐coupled receptors composed of taste receptor subunits T1R2 and T1R3. Beyond the tongue, STRs are expressed in the small intestine, brain, adipose tissue, and stomach, while they influence not only taste perception, but also digestive secretions, hormone release, and intestinal glucose absorption. Activation of STRs by sweet compounds can modulate the expression of glucose transporters, including sodium‐dependent glucose co‐transporter‐1 (SGLT‐1) and glucose transporter 2 (GLUT2), thereby influencing satiety and blood glucose regulation (Laffitte et al. 2014; Lee and Owyang 2017).

Given that individuals with obesity often exhibit a preference for sweet‐tasting foods, and considering the limited comparative data on the effects of different sweeteners, this study aimed to investigate the impact of natural MFE, synthetic sucralose, and sucrose at equivalent sweetness levels within the acceptable daily intake (ADI) range on body weight, adiposity, and glucose homeostasis in HFD‐induced obese C57BL/6 mice. Furthermore, we examined the potential involvement of intestinal STRs, GLUT2, and SGLT‐1 in mediating these effects.

## 2 Materials and Methods

### 2.1 Animals and Study Design

Five‐week‐old male C57BL/6 mice (BioLASCO, Taipei, Taiwan) were housed under controlled conditions and fed an AIN‐93 M‐based HFD (60% kcal from fat, 30% from carbohydrate, 10% from protein, MP Biomedicals, Irvine, CA) for 8 weeks to induce obesity. After obesity induction, mice were randomly assigned to one of four groups and provided drinking water containing either: distilled water (control), sucrose (266 g/L, MP Biomedicals), MFE (1.1 g/L containing 50.1% mogroside V; Huacheng Biotech, Hunan, China), or sucralose (0.36 g/L, Tate & Lyle, London, UK). The experimental dose was set at 50% of the sucralose ADI. Based on a mouse body weight of 30 g and water intake of 2.5 mL/day, this dose is equivalent to approximately 2.5 mg/kg/day in humans, applying the species‐specific dosage conversion factor (Nair and Jacob 2016). To maintain sweetness equivalence—accounting for sucralose being ~600 times sweeter than sucrose and Mogroside V being 150–250 times sweeter—the concentrations for MFE (containing ~50% Mogroside V) and sucrose were adjusted to 1.1 g/L and 266 g/L, respectively. The intervention continued for 8 weeks. Body weight, food intake, and water consumption were recorded three times per week. Oral glucose tolerance tests (OGTTs) were performed before and after the intervention. At the study endpoint, mice were sacrificed, and blood, organs, and small intestinal mucosa were collected for further analysis. All procedures were approved by the Institutional Animal Care and Use Committee of Taipei Medical University (LAC‐2021‐0132).

### 2.2 Oral Glucose Tolerance Test (OGTT)

OGTT was performed on half of the experimental animals (*n* = 5) following protocols adapted from Ryuk et al. (2019). To evaluate acute single‐dose effects, HFD‐induced obese mice were fasted overnight and orally administered a single dose (25 mg/kg body weight) of MFE or sucralose 30 min prior to a glucose load (2 g/kg). In contrast, the sucrose group received only the sucrose load (2 g/kg) at time 0 without a 30‐min preload. This was specifically intended to prevent an experimental ‘boost’ or premature elevation of blood glucose levels that would have interfered with the baseline (time 0) reading, given the caloric and glycemic nature of sucrose. This preload was intended to compare the acute glycemic modulatory effects of NNS of a standard glucose load given their negligible direct impact on blood glucose. However, because different carbohydrate loads were administered among the groups, this approach primarily allows for the assessment of the relative postprandial glucose responses associated with NNS consumption. Another limitation of this design is the absence of a non‐preload OGTT baseline, which precludes a definitive distinction between the acute effects of the sweetener and the independent glycemic response to the glucose load.

After the 8‐week intervention, a second OGTT was performed to evaluate chronic metabolic effects. Overnight‐fasted mice were orally administered glucose (2 g/kg body weight). Because a baseline OGTT without preload was not included, the influence of prior sweetener exposure on acute glycemic responses could not be fully distinguished. Blood samples were collected from the tail vein at 0, 15, 30, 60, 90, and 120 min after carbohydrate administration. Blood glucose concentrations were measured using a Contour Plus glucometer (Bayer, Wuppertal, Germany), and the area under the curve (AUC) was calculated using GraphPad Prism 8 (San Diego, CA, USA).

### 2.3 Biochemical Analysis

Blood was collected via cardiac puncture under isoflurane anesthesia. Serum levels of aspartate aminotransferase (AST), alanine aminotransferase (ALT), glucose, total cholesterol (TC), total triglycerides (TG), and high‐density lipoprotein cholesterol (HDL‐C) were measured using a Hitachi 7080 Automatic Biochemistry Analyzer (Tokyo, Japan). Low‐density lipoprotein cholesterol (LDL‐C) was calculated using the Friedeward formula: LDL‐C = TC − HDL‐C − TG/5.

Serum insulin (Mercodia, Uppsala, Sweden), adiponectin, and leptin (Biovendor LLC, Brno, Czech Republic) were quantified using commercial ELISA kits according to the manufacturers' protocols. In brief, serum samples were incubated with the specific primary antibody, followed by the addition of enzymatic substrates and the reaction mixture. The resulting colorimetric change was quantified by measuring the absorbance at 450 nm using a microplate reader. Target concentrations were subsequently determined by interpolation from the respective standard curves.

### 2.4 RNA Extraction and Quantitative Polymerase Chain Reaction (qPCR)

To evaluate the effects of sweeteners on intestinal glucose absorption, mRNA expressions of STRs and glucose transporters were determined. Total RNA was extracted from the duodenal mucosa using the RNeasy Mini Kit (Qiagen Ltd., Taipei, Taiwan). The RNA concentration and purity were assessed by measuring absorbance at 260 and 280 nm using a microplate reader (BioTek, Vermont, USA); only samples meeting purity requirements (A_260_/A_280_ ratio ~2.0) were used for subsequent analysis. Total RNA (2.5 μg) was reverse‐transcribed to complementary DNA (cDNA) using the RevertAid Reverse Transcriptase system with oligo(dT) primers and dNTPs. The reaction was incubated at 55°C for 60 min and terminated by heating at 70°C for 15 min. Quantitative PCR (qPCR) was performed using the QuantStudio 1 Real‐Time PCR System (Thermo Fisher Scientific, Cambridge, MA, USA) with specific primers and Maxima SYBR Green/ROX qPCR Master Mix. The thermal cycling conditions consisted of an initial step at 50°C for 2 min and 95°C for 10 min, followed by 40 cycles of denaturation at 95°C for 15 s and annealing/extension at 60°C for 1 min. All samples were analyzed in duplicate, and primer sequences are provided in Table S1.

### 2.5 Statistical Analysis

Data are presented as the mean ± standard deviation (SD). Adhering to the 3Rs principles for animal welfare, the sample size was minimized; the water control group consisted of *n* = 6 mice, and the treatment groups consisted of *n* = 10 mice/group. After confirming the normality of distribution using Shapiro–Wilk test, statistical comparisons were performed using Student's *t*‐test or one‐way analysis of variance (ANOVA) followed by Tukey's post hoc‐test for multiple comparisons. All statistical analyzes were conducted using SPSS version 19.0 (IBM, Armonk, NY). The area under the curve (AUC) for blood glucose was calculated using GraphPad Prism 8 (GraphPad Software, San Diego, CA, USA). Statistical significance was defined as *p* < 0.05.

## 3 Results

### 3.1 Body and Organ Weights, Food, and Water Intake

Based on an average water consumption and a mean body weight of approximately 40 g, the estimated daily intake was approximately 100 mg/kg/day for MFE and 47 mg/kg/day for sucralose. Although sucralose dose was slightly exceeded our initial projections (30 mg/kg/day), it remained within the established ADI range when adjusted to human equivalent dose. Following 8 weeks of sweetener administration, both MFE and sucrose significantly increased body weight compared with the water control. Notably, the sucrose and sucralose groups exhibited the most pronounced weight gains among the HFD‐fed obese mice (Table 1). Despite significantly higher food and water consumption compared to the control group, MFE‐treated mice exhibited no significant difference in body weight gain. Organ and tissue weights did not differ among groups, except for a lower relative kidney weight in the sucrose group. Collectively, these findings indicate that despite increased food and water consumption, mice in the MFE group exhibited body weight gain comparable to the water control group, whereas sucrose and sucralose further exacerbated weight gain in obese mice.

**TABLE 1:** Effects of sweeteners on body and organ weights, food and water intakes in mice fed with an HFD.

Group		Water control	MFE	Sucrose	Sucralose
Body weight					
Initial (g)		34.84 ± 2.99	37.30 ± 2.81	35.89 ± 3.39	34.67 ± 3.99
Final (g)		40.32 ± 1.50^a^	44.00 ± 2.95^b^	44.60 ± 2.63^b^	43.20 ± 2.73^ab^
Change (g)		5.48 ± 1.63^a^	6.70 ± 1.63^ab^	8.71 ± 1.68^b^	8.52 ± 1.97^b^
Average food intake (g/day)		2.36 ± 0.19^a^	2.98 ± 0.57^b^	2.19 ± 0.23^a^	3.13 ± 1.06^b^
Average water intake (mL/day)		2.20 ± 0.69^a^	3.63 ± 1.99^b^	3.93 ± 1.32^b^	5.22 ± 2.65^c^
Food efficiency ratio (%)^1^		3.89 ± 1.15^a^	3.85 ± 1.10^a^	6.77 ± 1.37^b^	4.66 ± 1.34^a^
Organ & tissue weights					
Heart	(g)	0.17 ± 0.05	0.17 ± 0.03	0.15 ± 0.02	0.15 ± 0.02
	(%)^2^	0.43 ± 0.13	0.38 ± 0.05	0.34 ± 0.05	0.35 ± 0.06
Liver	(g)	1.19 ± 0.18	1.21 ± 0.26	1.43 ± 0.30	1.19 ± 0.08
	(%)	2.95 ± 0.39	2.75 ± 0.56	3.19 ± 0.59	2.77 ± 0.25
Spleen	(g)	0.11 ± 0.05	0.10 ± 0.03	0.09 ± 0.03	0.10 ± 0.01
	(%)	0.27 ± 0.11	0.23 ± 0.06	0.21 ± 0.06	0.24 ± 0.04
Kidney	(g)	0.41 ± 0.08	0.42 ± 0.04	0.36 ± 0.05	0.39 ± 0.04
	(%)	1.01 ± 0.21^b^	0.95 ± 0.08^ab^	0.81 ± 0.12^a^	0.91 ± 0.12^ab^
White adipose tissue					
Perirenal	(g)	0.97 ± 0.25	1.16 ± 0.29	1.20 ± 0.28	1.19 ± 0.26
	(%)	2.41 ± 0.62	2.61 ± 0.56	2.69 ± 0.62	2.75 ± 0.61
Epidydimal	(g)	2.18 ± 0.33	2.03 ± 0.39	2.17 ± 0.43	2.01 ± 0.59
	(%)	5.44 ± 0.99	4.61 ± 0.90	4.90 ± 1.09	4.62 ± 1.24
Muscle					
Gastrocnemius	(g)	0.34 ± 0.07	0.36 ± 0.04	0.38 ± 0.06	0.34 ± 0.03
	(%)	0.87 ± 0.16	0.83 ± 0.11	0.84 ± 0.12	0.80 ± 0.11
Tibialis anterior	(g)	0.12 ± 0.02	0.13 ± 0.02	0.13 ± 0.02	0.13 ± 0.01
	(%)	0.30 ± 0.05	0.29 ± 0.06	0.29 ± 0.05	0.30 ± 0.03

*Note:* The animals were administered distilled water (control), MFE (1.1 g/L), sucrose (266 g/L), or sucralose (0.36 g/L) in their drinking water for 8 weeks to HFD‐induced obese mice. Following the intervention, the animals were sacrificed, and organs were collected and weighed. Data are presented as the mean ± SD. Intergroup differences were analyzed using one‐way ANOVA followed by Tukey's post hoc test. ^a–c^Data not sharing the same superscript significantly differ (*p* < 0.05).

^1^ Food efficiency ratio was calculated as body weight gain (g)/average food intake (g).

^2^ Relative organ weight (%) = organ weight (g)/final body weight (g) x 100%. HFD, high‐fat diet containing 60% kcal from fat; MFE, Monk fruit extract.

### 3.2 Serum Biochemical Measurements

No significant intergroup differences were detected in serum AST, ALT, insulin, triglycerides, LDL‐C, adiponectin, and leptin (Table 2). However, fasting blood glucose levels were significantly lower in the MFE group compared with the water control. In addition, MFE‐treated mice exhibited lower total cholesterol and HDL‐C levels relative to the sucrose group.

**TABLE 2:** Effects of sweeteners on blood biochemical measurements in obese mice fed with an HFD.

Item/group	Water control	MFE	Sucrose	Sucralose
AST (U/L)	127.05 ± 21.04	137.47 ± 43.78	160.12 ± 54.93	147.01 ± 49.05
ALT (U/L)	59.98 ± 26.64	91.26 ± 78.94	103.34 ± 75.70	97.15 ± 51.68
Glucose (mg/dL)	197.75 ± 9.09^b^	138.27 ± 28.75^a^	166.13 ± 35.25^ab^	144.77 ± 43.22^ab^
Total cholesterol (mg/dL)	126.73 ± 38.39^ab^	128.06 ± 35.22^a^	173.62 ± 42.51^b^	139.74 ± 29.84^ab^
Triglycerides (mg/dL)	33.98 ± 1.27	26.44 ± 11.08	32.40 ± 14.99	27.10 ± 12.04
HDL‐C (mg/dL)	98.75 ± 27.71^ab^	92.48 ± 26.59^a^	126.31 ± 24.30^b^	99.86 ± 23.12^ab^
LDL‐C (mg/dL)	21.18 ± 11.40	30.29 ± 10.06	40.83 ± 17.33	34.46 ± 10.46
Insulin (μg/L)	0.96 ± 0.71	1.13 ± 0.70	1.35 ± 0.87	1.67 ± 1.28
Adiponectin (ng/mL)	41.19 ± 17.80	39.16 ± 13.55	25.94 ± 10.53	26.67 ± 13.52
Leptin (μg/mL)	13.98 ± 14.63	17.17 ± 12.66	21.93 ± 13.38	15.55 ± 11.00

*Note:* The animals were administered distilled water (control), MFE (1.1 g/L), sucrose (266 g/L), and sucralose (0.36 g/L) in drinking water for 8 weeks in HFD‐induced obese mice, and blood samples were collected for analysis. Data are presented as the mean ± SD. Intergroup differences were analyzed by one‐way ANOVA followed by Tukey's post hoc test. ^a,b^Data not sharing the same superscript significantly differ (*p* < 0.05).

Abbreviations: ALT, Alanine transaminase; AST, Aspartate transaminase; HDL‐C, high‐density lipoprotein cholesterol; HFD, high‐fat diet containing 60% kcal from fat; LDL‐C, low‐density lipoprotein cholesterol; MFE, Monk fruit extract.

### 3.3 Oral Glucose Tolerance Test

**FIGURE 1:**
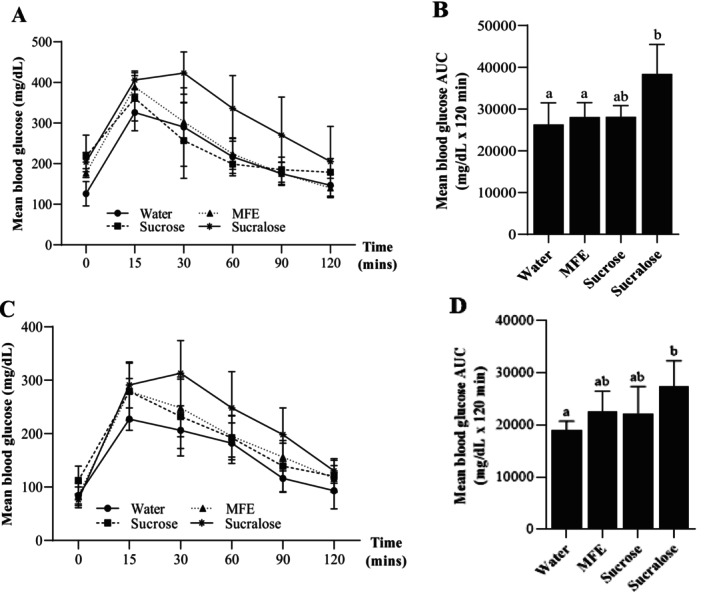
Effects of sweeteners on blood glucose levels (A, C) and area under the curve (AUC) of serum glucose (B, D) from oral glucose tolerance test (OGTT) in HFD‐induced obese mice. The OGTT was performed after a single dose (A, B) and after an 8‐week exposure (C, D) to water containing MFE (1.1 g/L), sucrose (266 g/L), and sucralose (0.36 g/L), respectively. Data are presented as the mean ± SD. Intergroup differences were analyzed by one‐way ANOVA followed by Tukey's post hoc test. ^a,b^Data not sharing the same superscript significantly differ (*p* < 0.05). MFE, Monk fruit extract. HFD, high‐fat diet containing 60% kcal from fat.

OGTTs were conducted to assess the effects of acute and chronic sweetener exposure on glucose homeostasis. In most groups, blood glucose levels peaked at 15 min post‐glucose load and returned to baseline by 120 min. In contrast, sucralose delayed the glucose peak to 30 min under both acute and chronic exposures (Figure 1A,C), leading to significantly higher AUC values (Figure 1B,D). Furthermore, a single dose of sucralose before glucose load elevated baseline blood glucose level at 0 min (204 ± 24 mg/dL vs. 126 ± 30 mg/dL in controls; Figure 1A). These results suggest that sucralose, whether acutely or chronically consumed, impairs glucose tolerance and may disturb postprandial glucose regulation in HFD‐induced obese mice.

### 3.4 Expression of Glucose Absorption‐Associated Genes in Duodenal Mucosa

**FIGURE 2:**
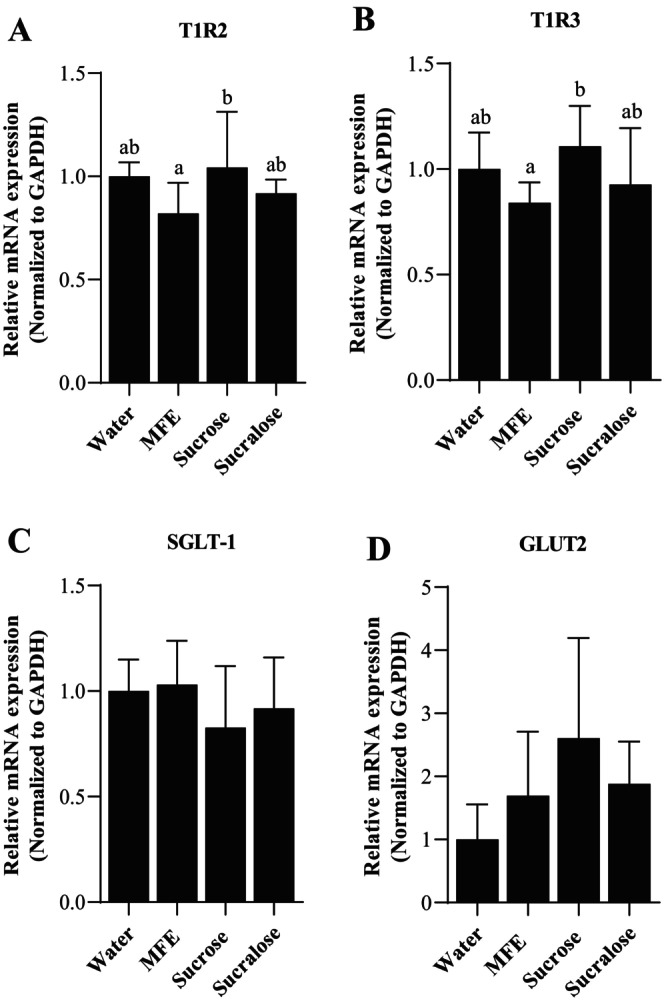
Effects of sweeteners on the mRNA expression of glucose absorption‐associated factors in the duodenal mucosa of obese mice fed an HFD. The animals were administered distilled water (control), MFE (1.1 g/L), sucrose (266 g/L), and sucralose (0.36 g/L) in drinking water for 8 weeks. After sacrifice, the duodenal mucosa was collected for analysis of the mRNA expression of T1R2 (A), T1R3 (B), SGLT‐1 (C), and GLUT2 (D) by qPCR. mRNA levels were normalized to the internal control GAPDH mRNA. Data are presented as the mean ± SD of duplicates. Intergroup differences were analyzed by one‐way ANOVA followed by Tukey's post hoc test. ^a,b^Data not sharing the same superscript significantly differ (*p* < 0.05). MFE, Monk fruit extract; T1R2, Taste receptor type 1 member 2; T1R3, Taste receptor type 1 member 3; SGLT‐1, Sodium‐dependent glucose cotransporter 1; GLUT2, Glucose transporter 2; GAPDH, glyceraldehyde‐3‐phosphate dehydrogenase.

To explore intestinal mechanisms underlying the differential effects of MFE and sucralose on glucose homeostasis, we examined duodenal mRNA expression of STR subunits (T1R2, T1R3) and glucose transporters (SGLT‐1, GLUT2). No significant differences were observed compared with water controls (Figure 2). However, MFE‐treated mice displayed lower T1R2 and T1R3 expression than sucrose‐treated mice (Figure 2A,B).

## 4 Discussion

This study demonstrates that daily consumption of MFE at doses within the ADI lowers fasting blood glucose levels and does not promote additional body weight gain in HFD‐induced obese mice, whereas sucralose exacerbates weight gain and impairs glucose tolerance. To our knowledge, this is the first study directly comparing natural and synthetic NNS, matched for equivalent sweetness and within human ADI levels, against sucrose in an obese animal model. The reduction in fasting glucose in MFE‐treated animals indicates a potential favorable metabolic consequence, distinguishing it from conventional sugars and some NNSs.

Sucrose and sucralose promoted greater weight gain, accompanied by increased caloric consumption through water and food intake, consistent with their obesogenic potential. In contrast, MFE‐treated mice consumed more food but did not gain additional body weight compared with controls, suggesting possible increased energy expenditure‐ and altered nutrient utilization‐related mechanisms. At the doses administered in this study (MFE, 100 mg/kg/day; sucralose, 47 mg/kg/day), the caloric contribution, if any, of both MFE and sucralose was negligible. Given that the caloric density of most glycosides is approximately 4 kcal/g, a 40 g mouse consuming 4 mg of MFE per day would receive only 0.016 kcal from the sweetener. Compared to the daily caloric intake from a high‐fat diet (approximately 12–15 kcal/day for an obese mouse), MFE accounts for less than 0.1% of total daily energy. Given that sucralose is non‐caloric, the observed weight gain in this group may be attributed to a compensatory increase in food intake, whereas the weight gain in the sucrose group was likely driven by an elevated feed efficiency ratio, because sucrose exhibited less food intake and water intake than the sucralose, yet with similar weight gain. Therefore, the observed metabolic effects and weight gain profiles in the MFE group are unlikely to be driven by direct caloric contribution, but rather by physiological responses to the sweetener itself or its metabolites. Previous studies reported weight reduction with higher doses of MFE (400~800 mg/kg/day) in obese mice (Li et al. 2020; Zhang et al. 2018), whereas our lower estimated dose (90–130 mg/kg/day) may explain the absence of significant weight loss. Moreover, adipose tissue weights were not altered, possibly due to depot‐specific lipid accumulation not captured by our analysis of only perirenal and epididymal fat (Kowalski et al. 2017; Mitsutomi et al. 2014).

A key finding is that MFE lowered fasting glucose, whereas sucralose impaired glucose tolerance in both acute and chronic administrations in obese mice. Although previous studies have suggested that mogroside‐rich extracts may modulate metabolic pathways, including hepatic AMP‐activated protein kinase (AMPK) activation, those were not directly evaluated in this study. AMPK activation may lead to decrease the mRNA expression of gluconeogenic enzymes, such as phosphoenolpyruvate carboxykinase (PEPCK) and glucose‐6‐phosphatase (G6Pase), thereby reducing hepatic glucose output (Klover and Mooney 2004; Li et al. 2020; Liu et al. 2019). In contrast, sucralose's adverse glycemic effects, as reflected by OGTT AUC and delayed glucose peaks. These findings are parallel to prior reports in rodents and humans demonstrating that sucralose may alter insulin sensitivity, incretin secretion or gut‐brain signaling (Chan et al. 2017).

The hypoglycemic effects of MFE or mogrosides may be mediated by multiple mechanisms, including enhanced hepatic glucose metabolism, increased insulin secretion and sensitivity, or modulation of inflammatory mediators (Ban et al. 2020; Liu et al. 2019; Zou et al. 2018). Intestinal sweet taste receptors (STRs) and glucose transporters (GLUTs) have also been indicated to be involved in NNS‐regulated postprandial glucose absorption. Margolskee et al. (2007) reported that NNSs, such as sucralose, saccharin, and ACE‐K, upregulate the expression of SGLT‐1 in wild‐type mice, but not in the STR knockout mice, thereby increasing intestinal glucose uptake. Similarly, Shi et al. (2021, 2024) demonstrated that sucralose enhances intestinal glucose absorption and elevates blood glucose levels in association with upregulated T1R2, T1R3, SGLT‐1, and GLUT2 expression. In the present study, we did not observe significant differences in duodenal STR or GLUT mRNA expression. Given higher STR and GLUT expression in jejunum and ileum (Sutherland et al. 2007), analyzes beyond the duodenum may provide further insights. Finally, recent evidence indicates that MFE and mogroside V can modulate gut microbial composition, improve intestinal barrier function, and attenuate endotoxemia‐associated insulin resistance. Although gut microbiota was not examined in the present study, its potential contribution to the observed metabolic effects cannot be ruled out (Qin et al. 2024; Wang et al. 2022; Xiao et al. 2021).

This study has several limitations that should be considered while interpreting the findings. First, male mice were selected in this study to minimize the potential confounding hormonal effects on metabolic parameters and glucose homeostasis. As sex differences in metabolic regulation, hormonal status, and responses to dietary interventions have been reported (de Souza et al. 2022), our findings may not fully reflect responses in female animals. Second, only a single dose of each sweetener was tested, and dose–response relationships were not explored. Third, expression of intestinal GLUT was assessed only at the mRNA level, with no protein confirmation, which limits interpretation of functional changes of the transporter proteins. Moreover, intestinal sampling was confined to the duodenum, whereas STRs and GLUTs are also abundantly expressed in the jejunum and ileum. Fourth, gut microbiota composition was not analyzed, despite the proposed role of microbiota‐mediated metabolism in the biotransformation of mogrosides. Fifth, the relatively small sample size used for the OGTT experiments may have reduced statistical power, although the results showed statistically significant differences. Besides, only preload OGTT experiment was performed, no baseline OGTT was obtained, and the use of sucrose as the carbohydrate challenge for the sucrose group, and these all weaken the interpretation the difference on glycemic responses. Finally, energy expenditure and substrate utilization were not directly measured, so the mechanisms responsible for the observed metabolic effects remain to be further elucidated.

Notwithstanding these limitations, the present study has several strengths. The study design enabled a direct comparison of natural and synthetic sweeteners under equivalent sweetness at ADI‐relevant doses conditions, thereby allowing a more rigorous evaluation of their relative metabolic effects under physiological conditions. In addition, both acute and chronic responses of glucose regulations were assessed, and molecular as well as metabolic endpoints were included to provide a broader view of treatment‐related effects. These findings suggest that MFE may exert more favorable metabolic effects than sucralose or sucrose and may warrant further investigation as a potential alternative sweetener for populations at risk of obesity and type 2 diabetes. Further studies should address long‐term outcomes, dose–response relationships, and microbiota‐mediated mechanisms to establish the translational relevance of MFE in human metabolic health.

In summary, our findings suggest that MFE, a natural sweetener, does not promote additional body weight gain and improves fasting blood glucose regulation without worsening glucose tolerance. In contrast, sucrose and the synthetic sweetener sucralose may exacerbate OGTT glycemic homeostasis under obesogenic conditions. Importantly, the glycemic effects of MFE and sucralose appear to be independent of duodenal glucose absorption.

## Author Contributions

**Jessika Woo Kar Man:** investigation, writing – original draft, methodology, formal analysis, software. **Rong‐Hong Hsieh:** supervision, project administration. **Yu‐Cih Huang:** investigation, formal analysis. **Sassy Bhawamai:** formal analysis, resources. **Yue‐Hwa Chen:** conceptualization, project administration, resources, supervision, writing – review and editing.

## Funding

This work was supported by the National Science and Technology Council (Grant NSTC 111‐2314‐B038‐003) and Taipei Medical University (Grant TMU111‐AE2‐I05‐1).

## Conflicts of Interest

The authors declare no conflicts of interest.

## Supporting information


**Table S1:** Primer sequences for RT‐PCR.

## Data Availability

The data that support the findings of this study are available on request from the corresponding author. The data are not publicly available due to privacy or ethical restrictions.
