# Supplementary material for: Mogroside‐Rich Monk Fruit Extract Improves Glycemic Control Without Promoting Additional Weight Gain Compared With Sucralose and Sucrose in High‐Fat Diet‐Induced Obese Mice
Source: Food Sci Nutr. 2026 Jul 14;14(7):e72121. doi: 10.1002/fsn3.72121 (PMC13369284; doi:10.1002/fsn3.72121)
Supplement: Supplementary file 1 — Table S1: Primer sequences for RT‐PCR. [file FSN3-14-e72121-s001.docx]

**Table S1** Primer sequences for RT-PCR.

| Gene | Forward (5→3) | Reverse (5→3) | Reference |
| --- | --- | --- | --- |
| GAPDH | TTCACCACCATGGAGAAGGC | GGCATGGACTGTGGTCATGA | ([Radbin *et al.*, 2014](#_ENREF_85)) |
| T1R2 | TGTGACGCCTGCATGAACAT | CTGAGTAGCTGCCATGGATA | ([Shi *et al.*, 2021](#_ENREF_93)) |
| T1R3 | CAGTCAAAGCATTGCTGCCT | ATAGCTGACCTGTGGCATGA | ([Shi *et al.*, 2021](#_ENREF_93)) |
| SGLT-1 | CCTCTCGGCCAAGAACATGT | TTCAGATAGCCACACAGGGTACA | ([Ota *et al.*, 2022](#_ENREF_77)) |
| GLUT2 | GTCCAGAAAGCCCCAGATACC | GTGACATCCTCAGTTCCTCTTAG | ([Gouyon *et al.*, 2003](#_ENREF_23)) |

GAPDH, glyceraldehyde-3-phosphate dehydrogenase; T1R2, Taste 1 receptor 2; T1R3, Taste 1 receptor 3; SGLT-1, sodium dependent glucose co-transporter-1; GLUT2, glucose transporter 2.

Gouyon, F., L. Caillaud, V. Carrière, C. Klein, V. Dalet, D. Citadelle, G. L. Kellett, B. Thorens, A. Leturque and E. Brot-Laroche (2003) Simple-sugar meals target GLUT2 at enterocyte apical membranes to improve sugar absorption: a study in GLUT2-null mice. *J Physiol* **552**(3), 823-832.

Ota, T., T. Ishikawa, T. Sakakida, Y. Endo, S. Matsumura, J. Yoshida, Y. Hirai, K. Mizushima, K. Oka, T. Doi, T. Okayama, K. Inoue, K. Kamada, K. Uchiyama, T. Takagi, H. Konishi, Y. Naito and Y. Itoh (2022) Treatment with broad-spectrum antibiotics upregulates Sglt1 and induces small intestinal villous hyperplasia in mice. *J Clin Biochem Nutr* **70**(1), 21-27.

Radbin, R., F. Vahedi and J. Chamani (2014) The influence of drinking-water pollution with heavy metal on the expression of IL-4 and IFN-γ in mice by real-time polymerase chain reaction. *Cytotechnology* **66**(5), 769-777.

Shi, Q., X. Zhu and S. Deng (2021) Sweet Taste Receptor Expression and Its Activation by Sucralose to Regulate Glucose Absorption in Mouse Duodenum. **86**(2), 540-545.
